# Association Between Earlobe Crease and the Metabolic Syndrome in a Cross-sectional Study

**DOI:** 10.4178/epih/e2012004

**Published:** 2012-08-31

**Authors:** Eun Hee Kang, Hee Cheol Kang

**Affiliations:** Department of Family Medicine, Yonsei University College of Medicine, Seoul, Korea.

**Keywords:** Earlobe crease, Metabolic syndrome, Risk factors

## Abstract

**OBJECTIVES:**

Several studies found a significant association between earlobe crease (ELC) and cardiovascular disease (CVD). Metabolic syndrome (MS) is a group of high-risk factors that are a collection of cardiovascular risk factors. Scant data are available about the relationship between ELC and MS. The purpose of the current study was to examine the correlation between ELC and MS.

**METHODS:**

A cross-sectional study was performed on 3,835 subjects (1,672 females, 43.6%) aged 20 to 79 years who visited a health promotion center. To increase the reliability of the diagnosis of MS, both the modified National Cholesterol Education Program Adult Treatment Panel III (NCEP ATP III) and International Diabetes Federation (IDF) criteria were applied. Independent association between ELC and MS was assessed using multiple logistic regression analysis after adjusting for confounding variables.

**RESULTS:**

The frequency of ELC was 20.89% and the prevalence of MS was 11.03% (NCEP criteria) and 9.75% (IDF criteria). The prevalence of both ELC and MS significantly increased with age. The modified Framingham risk score was significantly higher in subjects with ELC than without. After adjusting for conventional risk factors for CVD, the risk of MS increased significantly in the presence of ELC.

**CONCLUSION:**

The current study showed that the odds ratio for MS increased in the presence of ELC in Korean adults. ELC is an auxiliary indicator of MS, although prognostic value might be limited. Further studies are warranted to elucidate the clinical significance of ELC.

## INTRODUCTION

**Figure 1:**
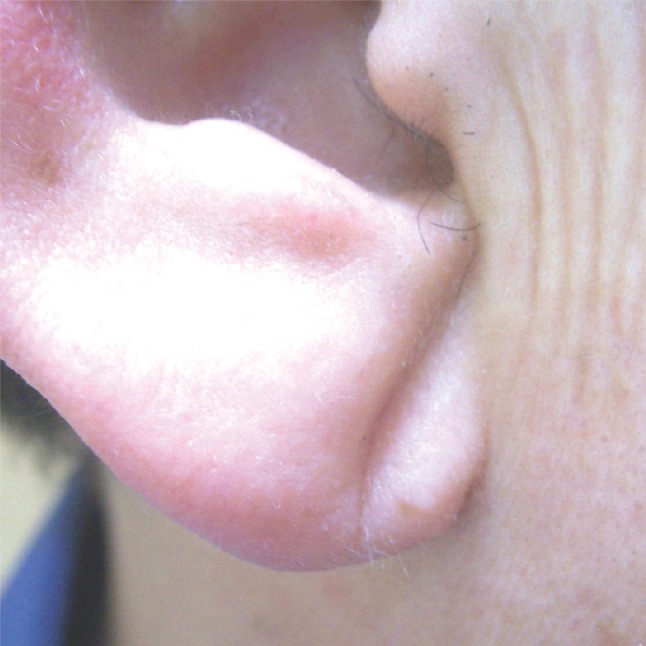
Typical shape of earlobe crease.

Diagonal earlobe crease (ELC) (Figure 1) is a diagonal wrinkle or deep furrow that extends from the external part of the external acoustic meatus to the bottom edge of the earlobe [1,2].

A relationship between the presence of ELC and cardiovascular disease (CVD)was first reported by Frank et al. [3]. Thereafter, several clinical studies, including autopsy-based [4] and histopathological examinations [5], confirmed that ELC is correlated with CVD [6,7]. In Korea, Park et al. [8] reported that ELC was an independent risk factor for hemorrhagic stroke. Celik and colleagues [9] found that ELC was correlated with carotid intima-media thickness (IMT), which has recently been used in the diagnosis of systemic atherosclerosis. These latter studies support the hypothesis that ELC was related with CVD.

It is widely known that metabolic syndrome (MS) is a global cardiometabolic risk factor for CVD [10]. It has also been shown that MS occurs due to accumulated risk factors for developing atherosclerosis [11-13]. For several years, MS has been regarded as a disease entity with well-known risk factors for developing CVD including hypertension, high low-density lipoprotein (LDL)-cholesterolemia, low high-density lipoprotein (HDL)-cholesterolemia, hyperglycemia, obesity, physical inactivity, and blood coagulation disorder [14], which are commonly found in modern society.

Although CVD, ELC, and MS might have a common pathophysiology such as atherosclerosis or vascular changes, no studies have reported a correlation between ELC and MS. We performed a cross-sectional study of a sample of the Korean population to evaluate the relationship between ELC and MS.

## MATERIALS AND METHODS

### Study subjects

This study was conducted on a total of 4,243 subjects (1,868 females, 2,375 males) aged between 20-79 years old who had received a standard health check-up at the Health Promotion Center of Severance Hospital between October 2007 and March 2008. ELC was defined as a diagonal wrinkle or deep furrow extending from the external part of the external acoustic meatus to the earlobe without a discontinuity. Unilateral and bilateral ELC were both considered to be ELC positive. ELC was measured by the same observer. Of the total subjects, 219 who had pierced ears (n=142) or showed an incomplete pattern of ELC (n=77) in whom a diagnosis of ELC might be confused, were excluded from analysis. Subjects with pre-existing CVD (n=189) including myocardial infarction, angina pectoris, stroke, transient ischaemic attack, carotid endarterectomy, and peripheral arterial occlusive disease, were also excluded, leaving 3,835 subjects that were enrolled in the study. The study was approved by the Institutional Review Board at Severance Hospital, Yonsei University College of Medicine in Seoul, Korea.

### Assessment of cardiovascular risk factors

In addition to medical history, personal history of smoking and family history of premature coronary artery disease (CAD) were examined in an interview. To increase the reliability of diagnostic criteria for MS, both the modified National Cholesterol Education Program Adult Treatment Panel III (NCEP ATP III) [15] and and International Diabetes Federation (IDF) definitions [16] were applied. Waist circumference was measured based on criteria for determining abdominal obesity in Koreans (males, ≥90 cm; females, ≥85 cm) [17]. The Framingham risk score (FRS), a differential scoring system based on the modified NCEP ATP III from 2002, was used to assess age, smoking, total cholesterol, HDL cholesterol, hypertension, and sex [10].

### Assessment of anthropometric and laboratory factors

Height and weight were measured without shoes in light indoor clothing to the nearest 0.1 cm and 0.1 kg, respectively. Body mass index (BMI) was calculated by dividing weight (kg) by the square of height (m^2^). In accordance with the guidelines of the National Institutes of Health (NIH), waist circumference was measured to the nearest 0.1 cm on a horizontal plane to the level directly above the iliac crest in an upright position. Blood pressure was measured using an automatic blood pressure monitor (ROLL STAND MMS-2000R, Mediana Co., Seoul, Korea) after a five-minute stabilisation. Subjects were not allowed to drink coffee or smoke cigarettes within 30 minutes following blood pressure monitoring. A sample of fasting venous blood was taken for the measurement of glucose, total cholesterol, HDL cholesterol, LDL cholesterol, and triglyceride. For venous blood samples, serum biochemical parameters were measured after a ten-hour fast. The blood samples were analysed using an automated chemistry analyzer (Hitachi 7600, Hitachi High-Technologies Co., Tokyo, Japan).

### Statistical analysis

To examine the distribution of statistical data, all continuous variables were expressed as mean and standard deviation (SD). Chi-square test was used for comparison of ELC, sex, past history of disease, and family history. Intergroup comparison of cardiovascular risk factors such as age, blood pressure, fasting blood sugar and BMI was made using 2-sample t-test. Following adjustment for cardiovascular risk factors including age, multiple logistic regression analysis was used to evaluate the effect of ELC on MS. The correlation between cardiovascular risk factors that had multicollinearity was also examined. Furthermore, to assess the significance of various models formed by various combinations, a likelihood ratio test and the Hosmer-Lemeshow test were performed. A p-value of <0.05 was considered statistically significant. All statistical analyses were performed using SAS version 9.1 (SAS Inc., Cary, NC, USA).

## RESULTS

### Baseline characteristics of the subjects

Baseline characteristics and assessment categories of 3,835 subjects (1,672 females, 43.60%) enrolled in the current study are summarised in Table 1. Except for a family history of CAD, all categories showed a significant difference depending on a MS diagnosis.

### Prevalence of MS and ELC in each age group

Of the 3,835 subjects, 11.03% were diagnosed with MS, according to the modified NCEP ATP III criteria. According to the IDF criteria, this figure was 9.75%. There was no significant difference in the prevalence of MS between the two criteria. Table 2 shows that the prevalence of MS significantly increased with age under both classifications (p<0.001).

Of the 3,835 subjects, 801 (20.89%) had ELC. Table 3 shows that the frequency of ELC increased with age (p<0.001). In particular, the frequency of ELC was significantly higher in males than females (p<0.001).

### Correlation between ELC and FRS

To test for a correlation between ELC and the modified FRS, the FRS was analysed according to a three-grade classification based on the percentile value of ten-year risk (Table 4). The presences of ELC in both male and female subjects showed a significant correspondence (p<0.001) to a higher FRS category.

### Independent relationship between ELC and MS

As shown in Table 5, the odds ratio (OR) for MS was relatively higher in the presence of ELC in both the modified NCEP ATP III and IDF criteria. The current study shows that the OR for MS was higher when subjects had ELC, after adjusting for age (Model 1). After adjusting for well-known major risk factors for CVD, including age (males, >45 yr; females, >55 yr), hypertension, smoking, obesity, dyslipidemia, diabetes mellitus, and a family history of premature CAD (Model 2), the risk for developing MS significantly increased in the presence of ELC for both criteria. Even after adjusting for the risk factors of coronary artery diseases defined by the NCEP ATP III (Model 3), the OR for males was 1.81-2.17 and the OR for females was 2.42-2.97.

## DISCUSSION

The current study shows that the presence of ELC is significantly associated with MS, independent of conventional CVD risk factors, in Korean adults without CVD. Subjects with ELC showed higher FRS than subjects without ELC.

The prevalence of ELC increased depending on age. Several studies have assumed that ELC and CVD were both part of the aging process [6,18]. In the present study, ELC was independently associated with MS after adjusting for age, and so it might be rash to regard ELC as a mere coincidence of the aging process.

Our results confirmed that the presence of ELC was significantly associated with MS independent of other confounding risk factors for MS. In the current study, the validity of each model and whether multicollinearity existed among variables were evaluated. A comparison of data explicability was made using a likelihood test between the models. This showed that Model 2 had the highest explicability. In Model 2, ELS was associated with MS after adjusting for age and other CVD risk factors in both sexes.

The FRS is a score-based tool that is widely used to predict atherosclerotic CVD [19]. In the current study, the percentile value of ten-year risk corresponding to the scores calculated in both males and females was analysed according to a three-grade classification and examined to determine whether it was correlated with ELC. This analysis showed that there was a significant correlation for both males and females (p<0.001). The prevalence of ELC was higher in the low-risk group than in the high-risk group of FRS category. This result might be caused by the low number of subjects enrolled in the high-risk group. The subjects with a past history of CVD were excluded and only those of good health were enrolled in the current analysis, so the actual number in the high-risk group was much lower than in the low-risk group.

To date, several hypotheses have been proposed to explain the possible mechanism for the correlation between ELC and CVD. First, the earlobe and heart are supplied with blood flow via an end artery with no collateral circulation, and second, both ELC and CVD are caused by the systemic loss of elastin and elastic fiber [5,20,21]. Based on reports that ELC was indicative of the severity of atherosclerosis [22], it has been demonstrated to have a correlation with carotid IMT [23-25], which has recently been used in the diagnosis of systemic atherosclerosis [9]. In the current study, ELC had a significant correlation with the FRS, which is an indicator for predicting CVD. Even after adjusting for factors that might affect CVD, the OR for MS was increased in the presence of ELC. This result suggests that ELC might be associated with MS in the subjects who did not have a CVD history.

It should be noted that the present study has several limitations. First of all no assessment of insulin resistance was concurrently made. The pathophysiology of MS is not clearly understood, although it is well established that insulin resistance is a very important factor. The current study did not consider such objective indicators as homeostasis model assessment of insulin resistance (HOMA-IR) index or euglycemic-hyperinsulinemic clamp study, which can be used to measure insulin resistance. Hence, further studies are warranted to identify the correlation between ELC and insulin resistance.

The recent understanding of the link between MS and atherosclerosis is that it may be more likely due to a chronic low-grade inflammatory status related to MS than to insulin resistance itself. The present study failed to include inflammatory markers such as hs-CRP. In future studies, the association between inflammatory markers and ELC should be assessed.

No correlation between the length ratio of ELC to earlobe and MS was assessed. Additionally, the difference between unilateral and bilateral ELC was not examined. Considering that no definite criteria for the length ratio of ELC to earlobe and those for the unilateral or bilateral presence of ELC have been established, we suggest that further studies are necessary to overcome these limitations.

The current study had a cross-sectional design and a causal relationship could not be confirmed. Long-term cohort studies are required to inclusively evaluate the course of insulin resistance, treatment response, and risk for developing CVD in MS patients who concurrently had ELC.

FRS has a trend which overestimates the CVD risk in Koreans, so this might affect the results.

Interpretation of ELC was performed by only one observer, and so the results of the present study may be biased.

CVD accounts for many deaths in Korea and around the world. Prediction and prevention of CVD are therefore significant factors in the clinical setting. In this regard, the current study is of great significance in that it identifies a correlation between MS, which is a group of high risk factors for CVD, and ELC. MS can confound the association between ELC and CAD, and so future studies of the ELC and CAD association should take into account the presence of MS.

## Figures and Tables

**Table 1:**
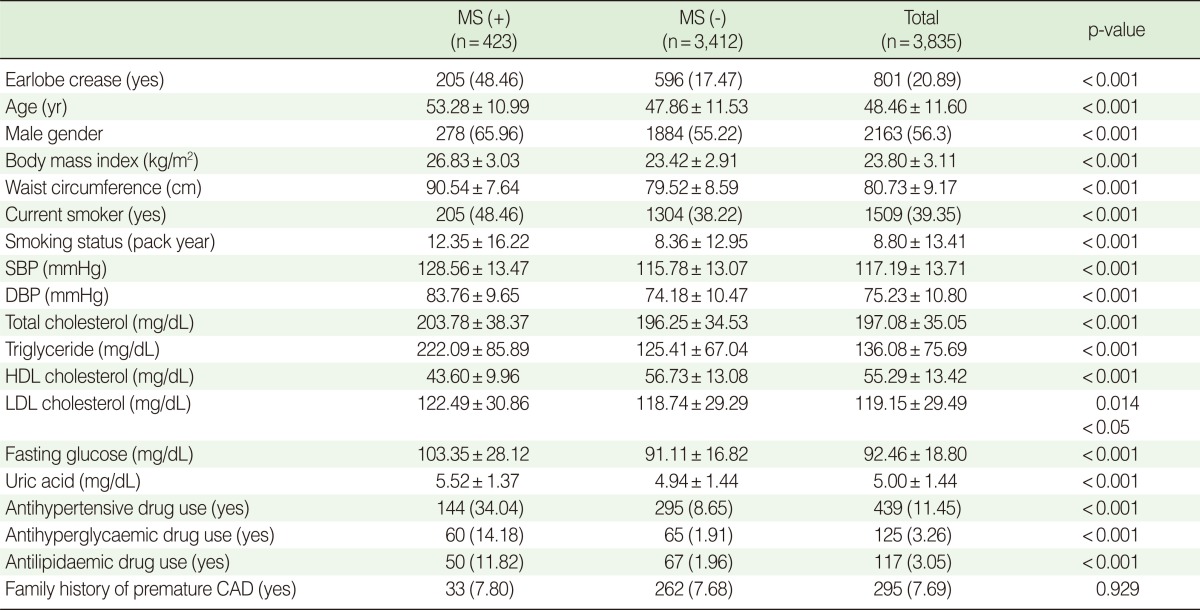
Baseline characteristics depending on MS (modified NCEP ATP III criteria)

Values are presented as number (%) or mean±standard deviation. MS, metabolic syndrome; SBP, systolic blood pressure; DBP, diastolic blood pressure; HDL, high-density lipoprotein; LDL, low-density lipoprotein; CAD, coronary artery disease.

**Table 2:**
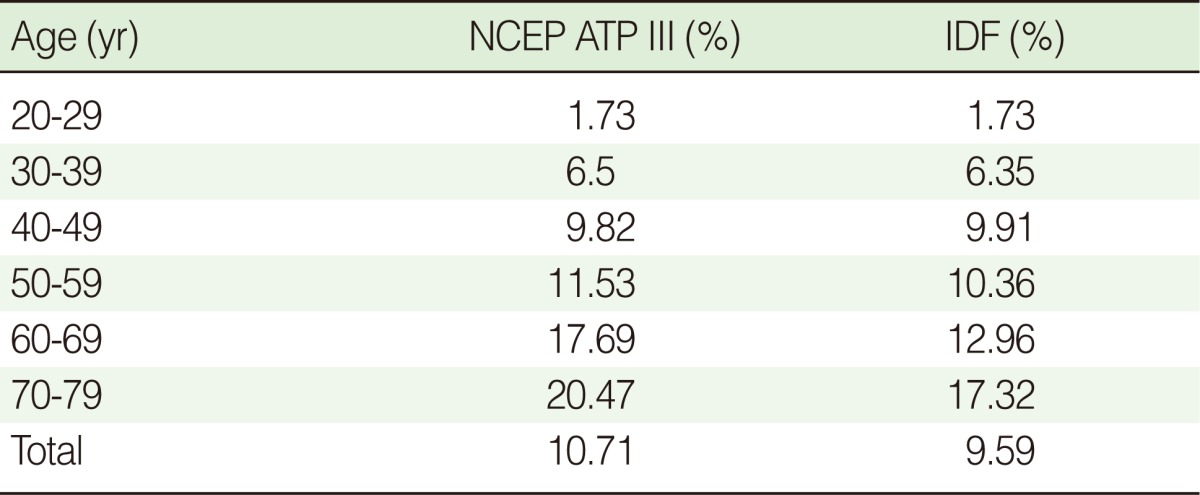
The prevalence of metabolic syndrome depends on age

NCEP ATP III, National Cholesterol Education Program Adult Treatment Panel III; IDF, International Diabetes Federation.

**Table 3:**
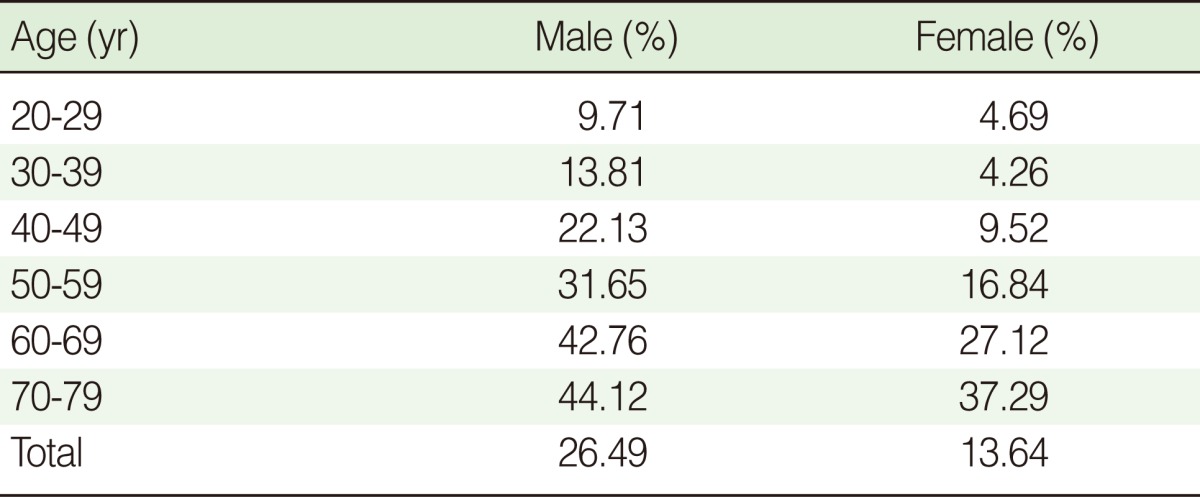
The prevalence of ELC depends on age

ELC, earlobe crease.

**Table 4:**
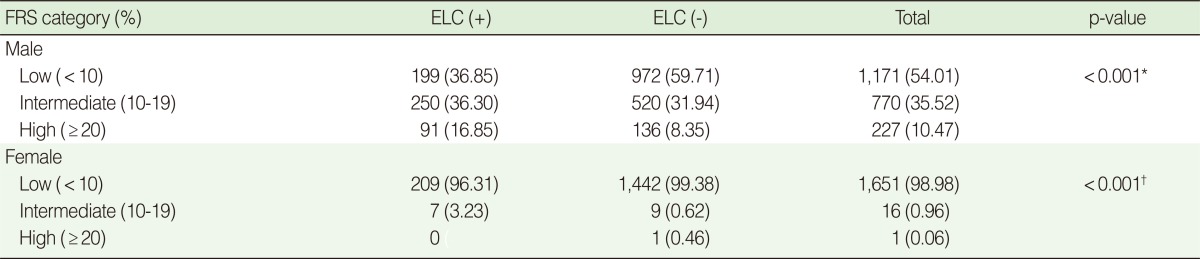
The modified FRS category depending on the presence of ELC in male and female subjects

Values are presented as number (%). FRS, Framingham risk score; ELC, earlobe crease; ^*^Chi-square test; ^†^Fisher's exact test.

**Table 5:**
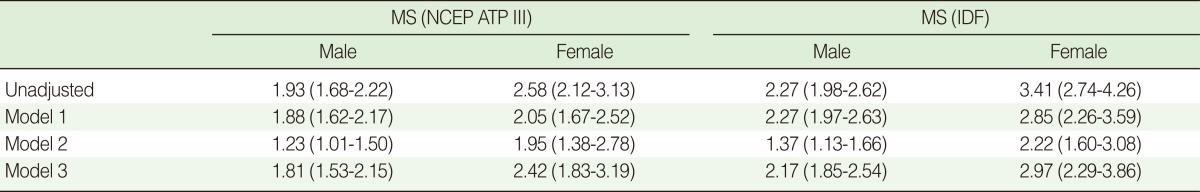
Independent association between ELC and MS after adjustment.

Values are presented as OR (95% confidence interval). Model 1: adjusted for age. Model 2: adjusted for age, hypertension, smoking, obesity, dyslipidaemia, diabetes mellitus, family history of premature coronary artery disease. Model 3: adjusted for age, hypertension, high-density lipoprotein cholesterol, family history of premature coronary artery disease. ELC, earlobe crease; MS, metabolic syndrome; NCEP ATP III, National Cholesterol Education Program Adult Treatment Panel III; IDF, International Diabetes Federation.
